# The Effectiveness of Parent Management Training—Oregon Model in Clinically Referred Children with Externalizing Behavior Problems in The Netherlands

**DOI:** 10.1007/s10578-016-0660-5

**Published:** 2016-06-15

**Authors:** Jill Thijssen, Gerko Vink, Peter Muris, Corine de Ruiter

**Affiliations:** 1 Department of Clinical Psychological Science, Maastricht University, P.O. Box 616, 6200 MD Maastricht, The Netherlands; 2 Department of Methodology and Statistics, Utrecht University, Utrecht, The Netherlands

**Keywords:** Parent management training, Externalizing behavior problems, Parenting practices

## Abstract

The present study examined the effectiveness of parent management training—Oregon model (PMTO) as a treatment for children with externalizing behavior problems in The Netherlands. Clinically referred children (*N* = 146) aged 4–11 years and their parents were partly randomized to either PMTO (*n* = 91) or Care As Usual (CAU; *n* = 55). Families were assessed at four time points: at pretreatment, and after 6, 12, and 18 months. Results showed that both PMTO and CAU were effective in reducing child externalizing behavior, parenting stress and parental psychopathology, with no significant differences between the two treatment conditions. PMTO and CAU interventions also produced some improvements in self-reported parenting skills, but not in observed parenting skills. According to the Reliable Change Index, 16.9 and 45.8 % of the children within the PMTO group showed full recovery or improvement in externalizing behavior, respectively, versus 9.7 and 42.8 % in the CAU condition. Finally, the effect size of PMTO on parent-reported externalizing behavior problems as found in the present study was comparable to that found in previous studies evaluating PMTO as an intervention for this type of child psychopathology.

## Introduction

Ineffective parenting is a well-established risk factor for the development of externalizing behavior problems in children [1–5]. The role of parenting in the emergence and maintenance of problematic child behavior is cogently explicated in Patterson’s Social Interaction Learning (SIL) model [1]. Briefly, the SIL model assumes that ineffective rearing practices have a direct detrimental influence on the behavior of the child, thereby hindering its healthy social-emotional development. More precisely, persistent coercive parenting—which is characterized by hostility and holding power over children via punitive or psychologically controlling means—can promote overt forms of externalizing behavior problems, such as noncompliance, temper tantrums, and verbal and physical aggression, which in turn are maintained by negative reinforcement of the parents [6]. Contextual factors, such as socio-economic disadvantage, parental psychopathology, and child temperament, are assumed to have a negative impact on parenting quality. For example, research has shown that parents of children with Attention Deficit Hyperactivity Disorder (ADHD) are less rewarding and consistent, display lower levels of warmth and involvement, and more often use physical discipline in comparison to parents of children without ADHD [7–9]. When children become more negative in their behavior, they are harder to discipline, which leads to parents using even more aversive strategies [10]. In this way, families become entangled in a downward spiral of negativity.

The antisocial behaviors acquired at home also tend to generalize to other social settings, such as school and sporting clubs [3]. Within the peer group, the antisocial behavior can lead to rejection by normal, prosocial peers. In turn, this can lead to associations with deviant peer groups [10–12] in which it pays off to show negative behaviors like lying, stealing, and vandalism [13]. However, parents also make a contribution to such deviant behavior by poor monitoring of the whereabouts and behaviors of their children outside the home environment. It enables youngsters to wander away from home and to engage in, for example, drug use and criminality [14]. These antisocial behaviors in childhood may take the form of an Oppositional Defiant Disorder (ODD) or Conduct Disorder [CD; 15], which have been shown to be possible precursors of Antisocial Personality Disorder in adulthood [16].

The fact that externalizing behavior problems in children can have significant negative long-term consequences, underlines the importance of early intervention programs. Many of these programs focus on the improvement of parenting practices and there is indeed evidence showing that the enhancement of positive and more effective parenting is an important mechanism that promotes children’s prosocial behavior [11, 17–20]. A good example of an intervention that is based on the key principles of the SIL model is parent management training—Oregon model (PMTO). The program is especially developed for the parents of children between 4 and 12 years of age showing the severe behavior problems associated with ODD or CD and aims to teach parents how to reduce coercive parenting practices and to replace these with five effective parenting practices: encouragement (i.e., stimulation of prosocial behaviors in the child by using scaffolding techniques and positive reinforcement), effective discipline (i.e., consistent use of mild sanctions like giving a time out), monitoring (i.e., knowing the child’s friends and keeping track of its activities), problem solving (i.e., responding effectively to rule-breaking behaviors and settling arguments with the child), and positive involvement [i.e., giving love and warm attention and engaging in fun activities with the child [18, 21].

Initial studies conducted in the Unites States (US) have demonstrated that PMTO is an effective intervention for reducing externalizing child behavior problems [e.g., 22, 23]. For instance, in the study by Forgatch and DeGarmo [18], 238 recently divorced mothers were randomly assigned to PMTO or a no intervention control condition. After 12 months, it was found that in the PMTO condition the effective parenting practices had significantly improved compared to the control condition. At a long term follow-up, 9 years after the PMTO intervention, there was still a significant difference between the boys in the PMTO condition and the control group with the former showing lower levels of delinquency, criminal activities, and convictions [13]. Furthermore, PMTO has also been shown to be effective in newly formed families consisting of biological mothers and stepfathers: again, parenting practices improved and behavior problems of the child decreased, as compared with newly formed families who did not receive an intervention [24]. Finally, in foster families, researchers found a success rate of permanent placements of 90 % for PMTO versus 64 % for Care As Usual (CAU) at an assessment which took place at 24 months after the interventions. PMTO was also significantly associated with reductions of stress for both the children and the foster parents [25].

The first randomized controlled trial (RCT) on the effectiveness of PMTO conducted outside of the US was completed in Norway, in which 112 clinically referred boys and girls aged between 4 and 12 years were randomly assigned to either PMTO or CAU [20]. Results indicated that PMTO was superior to CAU on the post-treatment outcome measures relating to effective discipline, obedience of the child, child-initiated negative behaviors and externalizing behavior problems. The effect of PMTO appeared to be moderated by the age of the child: that is, the intervention proved to be more effective in children below 8 years of age than among older children [20]. Further, at a 1-year follow-up, the differences between PMTO and CAU on child behavior problems and parental discipline were no longer significant [26]. A highly similar RCT was conducted in Iceland by Sigmarsdóttir et al. [27], who also allocated clinically referred children with behavior problems aged 5–12 years (*N* = 102) to either PMTO or CAU. PMTO was found to be more effective than CAU in improving general child adjustment post-treatment, although the only significant effect was documented for social skills. Surprisingly, this study did not obtain support for the idea that PMTO would have a positive effect on parenting skills [28].

Although PMTO is proven to be effective in the US, Norway and Iceland, this does not necessarily guarantee that this intervention also works in other countries. Therefore, the present study evaluated the effectiveness of PMTO in The Netherlands. One-hundred-and-forty-six families of clinically referred children with externalizing behavior problems aged between 4 and 11 years were included. The majority of the children (*n* = 96) was randomly assigned to either PMTO or CAU (in two treatment centers, such randomization was not possible as they only offered one of these interventions). Effects of PMTO and CAU were examined by means of measures of child externalizing behavior, parenting skills, and parental stress and psychopathology, which were administered at baseline, and three follow-up measurements after 6, 12, and 18 months. Parents’ treatment satisfaction was also evaluated after 6, 12, and 18 months. In addition, effect size and clinically significant change in children’s externalizing behavior problems was examined and compared across both treatment conditions, and several possible moderators of the effects produced by PMTO were explored (i.e., child, parent, and family variables). The following hypotheses were tested: (1) PMTO will result in greater improvements of children’s behavior problems, parenting skills, and parental stress and psychopathology than CAU; (2) PMTO will be associated with higher treatment satisfaction of parents as compared to CAU; and (3) PMTO will show a greater proportion of clinically significant change than CAU. With regard to moderator effects, predictions were less obvious, although it can be hypothesized that PMTO is more effective in families displaying the characteristics that are the target of this intervention (i.e., poor parenting skills) or that facilitate the application of the newly acquired skills in daily life (e.g., higher educational level of parent).

## Method

### Participants

Participants were 146 children and their parents, who were recruited at five child service agencies across The Netherlands. Of these children, 104 (71.2 %) were boys and 42 (28.8 %) were girls. At baseline, the age of the children ranged between 4 and 11 years, with a mean age of 7.13 years (*SD* = 1.75). Based on the Diagnostic Interview Schedule for Children [DISC; 29], 75.4 % of the children met the DSM diagnostic criteria for ADHD, 67.3 % for ODD, and 11.6 % for CD. There were 13 children who did not meet the full criteria of these disorders, but these children also displayed elevated levels of externalizing problems and hence qualify for a disruptive behavior disorder-not otherwise specified. The mean age of the main caregiver was 37.39 years (*SD* = 8.09). The vast majority of the main caregivers was female (90.5 %), had the Dutch nationality (89.7 %) and was employed (76.0 %). One-hundred-and-thirteen of the 146 main caregivers (77.4 %) were living with a partner, which in the majority of the cases (*n* = 106) was the other biological parent of the child. Five of the main caregivers were the adoptive parent of the child. These family characteristics are representative for the Dutch population. In Table 1, demographic characteristics are reported for the PMTO and CAU group separately. There were no significant differences between the children in both intervention groups. It should be noted, however, that the difference between treatment conditions in the presence of ADD/ADHD was almost significant: somewhat more children met the criteria for this disorder in the CAU condition as compared to the PMTO condition.

**Table 1:** Demographic data and significant differences for PMTO and CAU families

	PMTO (*n* = 91)	CAU (*n* = 55)	*p*
	*M* (*SD*) or %	*M* (*SD*) or %	
Child age at baseline	7.2 (1.81)	7.0 (.22)	.38
Parent age at baseline	38.7 (1.27)	37.1 (1.25)	.49
Child is a boy	71.7	70.4	.85
Main caregiver is female	88.0	85.2	.10
Dutch nationality	87.4	93.0	.53
Two parent household	78.3	75.9	.86
Biological parent	90.2	95.2	.93
Educational level			.26
High school	22.2	37.2	
IVE	36.3	32.0	
HVE	24.6	12.6	
University	11.1	9.4	
Child IQ	100.4 (1.48)	99.1 (1.98)	.59
ADD/ADHD	72.4	80.6	.06
ODD	65.2	70.9	.50
CD	10.5	13.3	.37

*IVE* intermediate vocational education, *HVE* higher vocational education, *ADD/ADHD* attention deficit disorder/attention deficit hyperactivity disorder, *ODD* oppositional defiant disorder, *CD* conduct disorder

To be included in the study, the child had to reveal a *T*-score of 60 or higher on the externalizing subscale of the Child Behavior Checklist [CBCL; 30, Dutch version: 31] and the child had to be residing at home with at least one biological or adoptive parent. Exclusion criteria were: severe intellectual disability or psychopathology of the parent(s) as this that would interfere with participation in treatment, sexual abuse in the family, and a child IQ lower than 70. These exclusion criteria were assessed during intake. Parents were asked whether they were capable to attend weekly therapy sessions. When there was a suspicion of severe substance use problems or sexual abuse, this was further investigated. In our sample, 19 children had an IQ lower than 85.

### Procedure

Families were included in the period between June 2009 and January 2014. As soon as families were referred to the child service agency, it was checked whether they met the inclusion criteria for the study. Families who met the criteria received information about the study and its procedure and were invited to participate. When parents agreed, they were asked to give their written consent. The study protocol was approved by the Medical Ethics Committee of Maastricht University Medical Centre.

Allocation to the treatment conditions (PMTO and CAU) was random at three of the five child service agencies. At the other two agencies no randomization took place. After the study had started, one child service agency decided it would no longer offer CAU and thus only recruited families for the PMTO condition. The fifth agency was specifically included in the study to compensate for the missing CAU families, but unfortunately this agency was less successful in recruiting participants for the study. Eventually, this resulted in unequal sample sizes for the two treatment conditions, with 94 families receiving PMTO and 61 families receiving CAU. In the PMTO condition, 17 families (18 %) dropped out, of which two families never started. In the CAU condition, 10 families (16 %) dropped out of which seven families never started. No data could be collected for the families that never started. These families were discarded from the data analysis, leaving a final sample of 91 PMTO families and 55 CAU families.

**Fig. 1:**
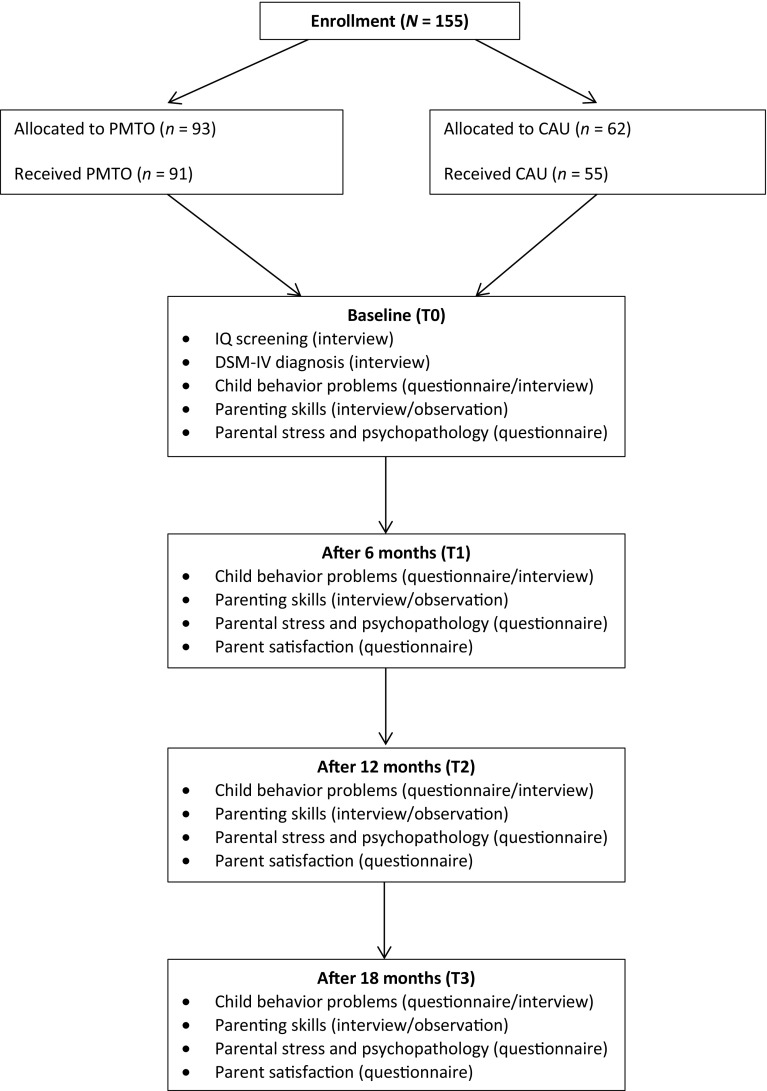
Flowchart of the present study. *PMTO* Parent management training—oregon model, *CAU* Care as usual

Figure 1 gives an overview of the assessments that were carried out during the course of this study. As can be seen, assessments were performed at four time points: at baseline (T0), and at 6-months (T1), 12-months (T2), and 18-months (T3) follow-up. IQ tests, interviews and video observations were mostly conducted at the child service agency (with a few exceptions at the families’ home), while questionnaires were administered to parents and teachers through a web-based system that could be approached by a computer in the home or agency environment. The assessments were conducted by trained research assistants who were not involved in the treatment of the families. The parent questionnaires and interviews were completed by the main caregiver, who was the parent who spent most time with the child. If present in the child’s family, the second caregiver was also assessed during the video observations. The number of families in which a second caregiver was present varied across the time points. Both caregivers were encouraged to attend the assessment sessions. However, due to, for example, work obligations, this was not always possible. The second caregiver was present in 64 families at T0, 40 families at T1, 32 families at T2, and 35 families at T3. Participating families received a small financial compensation in the form of gift vouchers for the three follow-up assessments (i.e., €10 at T1; €20 at T2; and €30 at T3).

The five child service agencies were independent therapy clinics for children. Therapists involved in the present study were all licensed clinicians working at the participating agencies. The researchers were not involved in the treatment of either PMTO or CAU. Therapists providing PMTO within the context of this study were not allowed to give CAU to families included in the study. In addition, CAU therapists were not allowed to be trained or to be in training as a PMTO therapist.

### Assessment

#### Descriptive Characteristics

##### IQ Measurement

To have an indication of the intellectual abilities of the children, a standardized IQ test was administered. When children were younger than 6 at baseline, the complete Wechsler Preschool and Primary Scale of Intelligence, 3rd edition [WPPSI-III; 32] was used for this purpose. For older children, the short form of the Wechsler Intelligence Scale for Children, 3rd edition [WISC-III; 33] was employed, which consists of two verbal (i.e., similarities and vocabulary) and two performance (i.e., picture arrangement and block design) subtests. The Dutch versions of the WPPSI-III and the WISC-III are both reliable and well-validated instruments that were current at the time that the study was conducted [34–36].

##### DSM-IV Diagnosis

The *Diagnostic Interview Schedule for Children* [DISC-IV; 29] is a highly standardized interview schedule to identify the presence of DSM-IV diagnoses in children. For the present study, the Disruptive Disorder module (module E) was used, which assesses three disorders: ADHD, ODD, and CD. The interview was administered and scored by trained clinicians.

#### Treatment Outcome Assessment

##### Child Behavior Problems

The *Child Behavior Checklist* (CBCL) and *Teacher Report Form* (TRF) are widely used rating scales for assessing behavioral and emotional problems of children aged 6–18 years [30],Dutch version: [31]. Each scale consists of 120 items that are scored on a 3-point Likert scale (0 = not true; 1 = somewhat or sometimes true; 2 = very or often true). Items can be allocated to narrow-band or DSM-based scales which represent specific types of problems (e.g., somatic complaints, social problems, affective problems, and conduct problems), or to the more general broad-band scales of internalizing (emotional) and externalizing (behavioral) problems, which can also be combined to a total score. For children aged 4 or 5 years, the 1.5–5 year version of the CBCL was used. The CBCL and TRF are widely used instruments that have been demonstrated to possess good reliability and validity [30, 31]. In the present study, the internalizing, externalizing, and total problems scores were used, which in the current study at baseline had Cronbach’s alphas of respectively .83, .84, and .90 (CBCL) and .84, .93, and .96 (TRF).

The *Parent Daily Report* (PDR) is a reliable 34-item checklist covering the range of mild (e.g., complaining) to severe (e.g., stealing) problem behaviors [23]. The checklist is first administered face-to-face with the main caregiver to assess which specific problem behaviors of the child have occurred during the past 6 months. Next, these items are administered again via telephone on three consecutive days to examine whether these behaviors have occurred during the past 24 h. The number of symptoms endorsed on the three consecutive days is summed to obtain the total PDR score. The internal consistency coefficient of the PDR at baseline was .80.

##### Parenting Practices

The Dutch translation of the *Caregiver Wish List* [CWL; 37, 38] is an interview-based instrument consisting of 53 items questioning the parent about his/her parenting skills. The interviewer reads the questions to the parent, who has to indicate the most applicable response option using a 5-point Likert scale. Items are allocated to six domains of parenting skills: providing direction and following up (4 items), encouraging good behavior (5 items), discouraging undesirable behavior (24 items), monitoring activities (13 items), connecting positively with child (3 items), and problem solving orientation (4 items). Each domain score should be regarded as a dimension with weak parenting skills on one end and strong parenting skills on the other. In the current study, the reliability of the CWL was not particularly strong: Cronbach’s alphas of various subscales were .22 for providing direction and following up, .44 for encouraging good behavior, .80 for discouraging undesirable behavior, .50 for monitoring activities, .55 for connecting positively with child, .37 for problem solving orientation, and .76 for the CWL total score.

*Structured Interaction Tasks* (SITs) were used to observe the parenting behaviors during a series of seven structured tasks that had to be performed by parents and their child: planning a fun activity for the weekend (3 min), problem solving, in which the family members discuss a topic chosen by the parent where they regularly have arguments about (5 min), drawing a picture of their own house (7 min), a snack-break to observe the family members when they do not have an assignment (5 min), problem solving for a topic chosen by the child (5 min), teaching tasks, which consist of two homework-like assignments of which one is slightly difficult for the child’s age and/or intellectual ability to evoke frustration (9 min), and monitoring, in which parents had to interview their child about a moment when there was no supervision by an adult (5 min). The SITs are designed to elicit parenting practices. The second problem solving task and the teaching tasks are performed with the main caregiver. When present, the drawing of a house is performed with only the second caregiver.

The tasks were videotaped and later coded by three trained, independent raters (psychology Master’s students) using an adapted version of the Coder Impressions developed by researchers at Oregon Social Learning Center [39]. Briefly, the videotaped SITs were employed to score a number of items referring to domains of effective parenting behaviors of positive involvement (12 items), encouragement (20 items), problem solving (27 items), discipline (25 items), and monitoring (5 items) as well as coercion (16 items), child compliance (8 items), and interpersonal atmosphere (24 items). For each SIT domain, item scores are accumulated, with higher scores reflecting better parenting skills. Cronbach’s alphas were poor for monitoring (mothers .28; fathers .06) and coercion (mothers .62; fathers .48). The other parenting dimensions had good internal consistency (range .74–.91).

All videotapes were coded by three trained and calibrated psychology graduate students blind to treatment condition and assessment point (i.e., T0, T1, T2, or T3). However, due to comments made by the parents or the experimenter during the interaction tasks, it could not always be prevented that the coders were aware of treatment condition or assessment point. Two independent raters coded a random selection of 103 SITs (29.4 % of the coded SITs). Interrater reliability was examined by means of a two-way mixed, consistency, average-measures Intraclass Correlation Coefficient [ICC; 40]. As can be seen in Table 2, the ICC’s for the parenting dimensions were fair to excellent, with the exception of ICC’s for discipline and compliance, which were poor (ICC < .40).

**Table 2:** Intraclass correlation coefficient for subscales of coded structured interaction tasks

	Main caregiver	Nonmain caregiver	Child
Positive involvement	.64	.45	
Positive reinforcement	.57	.61	
Problem solving	.80	.79	
Discipline	.10	−.88	
Monitoring	.47	.64	
Coercion	.51	.66	
Interpersonal atmosphere	.50	.62	.83
Compliance			.06

Two-way mixed, consistency, average-measures Intraclass Correlation Coefficient was used

##### Parental Stress and Psychopathology

The *Nijmeegse Ouderlijke Stress Index* [NOSI; 41] is an adaptation of the Parenting Stress Index (Abidin 1983) and measures stress experienced by parents in the relationship with their child. The NOSI comprises 123 items that have to be rated on a 5-point Likert scale (1 = strongly disagree, 5 = strongly agree). The items refer to parent (e.g., competence, social isolation, health, relationship with spouse) as well as child (e.g., hyperactivity, demandingness, mood) characteristics. Ratings on all items can be summed to create a total stress score, with higher scores reflecting higher levels of perceived stress by the parent. The NOSI has adequate reliability and validity [41]. In the present study, Cronbach’s alphas were .93 for the child domain, .94 for the parent domain, and .96 for the total score.

Psychological symptoms of the parents were measured by the Dutch version of the *Symptom Checklist*-*90 Revised* [SCL-90-R; 42]. This version of the questionnaire is based on the SCL-90-R of Derogatis [43]. The 90 items are rated using a 5-point scale (1 = no problem to 5 = very serious) to indicate the extent to which the parent has experienced the listed symptom during the previous week. In contrast to the original version of the SCL-90-R, the Dutch version comprises eight instead of nine subscales: Anxiety, Agoraphobia (in original version: Phobic Anxiety), Somatic Symptoms, Depression, Inadequacy of Thinking and Acting (in original version: Obsessive–Compulsive), Distrust and Interpersonal Sensitivity, Hostility, and Sleeping Problems. In the Dutch version of the SCL-90-R, the subscales Interpersonal sensitivity and Paranoid ideation (and three items from the original Psychoticism subscale) are combined into Distrust and Interpersonal Sensitivity due to insufficient discrimination between these dimensions [42]. Higher scores on the SCL-90-R indicate more serious psychopathology. In the present study, only the total score was used, which had an internal consistency coefficient of .97 at baseline.

##### Parent Satisfaction

The short form of the Working Alliance Inventory [WAI-S; 44] was used to assess the quality of the parent-therapist alliance. The WAI-S comprises 12 items that can be allocated to three subscales of four items each: (a) agreement between parent and therapist on the goals of the therapy; (b) agreement that the tasks of the therapy will address the parent’s problems, and (c) the quality of the bond between the parent and the therapist. Normally, the items of the WAI-S are rated using a 7-point Likert scale. However, in the present study, a 5-point Likert scale was used for practical reasons. Ten items are positively worded and two items (items 4 and 10) are negatively worded. The scores on the negatively stated items are recoded, so that all scores can be summed to obtain a total score. Higher total scores indicate a better parent-therapist working alliance. In the present study, Cronbach’s alpha for the total score was .71. Eventually, only the scores on T1 were used in the analyses.

#### Interventions

##### PMTO

PMTO is a therapy consisting of weekly sessions in which the therapist works with the parent(s) of one family. The children are not present during these sessions. The PMTO therapy aims to replace parents’ coercive parenting practices by the five effective parenting practices as defined by the SIL model. Role play is an important mechanism in the PMTO sessions to teach and extensively practice these effective parenting skills. The therapist uses the role play to demonstrate good and bad examples and to determine which parenting skills need extra attention [1]. As soon as the parent has sufficiently practiced the parenting skill, the therapist and parent choose a specific situation for the next week during which the parent will try to apply the newly acquired skill at home. In between sessions, the therapist calls the parent for support and to answer questions. Usually, the therapy takes place at the agency, but occasionally sessions are given at the parents’ home as well. Treatment duration depends on the family’s needs and progress throughout the therapy, but typically takes between 15 and 25 weekly sessions. Parents in the present study received PMTO from 25 certified therapists. All therapists had completed the full PMTO training program of approximately 24 months. During this training period, therapists had to treat at least three families with PMTO before they were allowed to take part in the official PMTO certification procedure. This procedure involved treating another family with PMTO. On the basis of videotaped sessions of this therapy, it was determined whether or not the therapists received their license to carry out PMTO in clinical practice. Following the completion of the training program, therapists were regularly monitored on their treatment fidelity, leading to annual recertification of their license. The association between treatment integrity and treatment outcome is addressed in a separate study [45]. Of the children for which medication use was documented (*n* = 66), 21.1 % used additional ADHD medication.

##### CAU

CAU-treatments were treatments that were available at the child service agencies for children with externalizing behavior problems and included family therapy (*n* = 31), psychiatric intensive home care (*n* = 10), parent therapy (*n* = 9), or other treatments (*n* = 6). In 9 CAU-families (17.6 %), children received ADHD medication in combination with one of the mentioned therapies. Two families in the CAU condition received more than one treatment, which explains the higher number of CAU therapies than CAU families.

#### Missing Data

The percentage of missing values in the dataset ranged from 0 % for the demographic variables to approximately 50 % for scores on the TRF. For many variables the missing values could be considered as bonafide because no score can be observed if the variable is not applicable (e.g., no observations for the second caregiver when only the main caregiver was present).

We applied an intention-to-treat design by conducting multiple imputation [46] to handle the missing data through a chain of conditional regression models [fully conditional specification; 47]. We used predictive mean matching [PMM; 48, 49] for the scale variables, a custom version of PMM for scale variables that contain bonafide missings, logistic regression for dichotomous variables and polytomous regression for ordered categorical data. All computations were carried out with Mice [50] in R [51], with 150 iterations for the algorithm to converge and 25 multiply imputed datasets, using available and custom imputation routines in Mice. The outcomes over the 25 datasets were combined into a single inference using Rubin’s rules [46, p. 76].

### Analytic Strategy

Overall effectiveness was examined for all outcome measures by using repeated measures ANOVA, with time (outcome measures at T0 through T3) as the within subjects factor and treatment condition (PMTO vs. CAU) as the between subjects factor. Because previous research found that child age and gender were significantly associated with treatment outcome [20, 28], we checked whether these variables had any influence on the outcome measures in our study. When this appeared to be the case, we controlled for the pertinent variable by performing an ANCOVA. An ANCOVA was conducted for the variables CBCL internalizing behavior problems, SIT interpersonal atmosphere of the child, and the NOSI child domain. To calculate effect sizes, the following Cohen’s d formula was used: Cohen’s.

$$d = \frac{{M_{1} + M_{2} }}{{SD_{pooled} }}$$

Furthermore, clinically significant change in externalizing behavior problems was examined using the Jacobson-Truax Reliable Change Index (RCI), since this is the most widely-used and recommended method [52, 53]. This method consists of two steps. First, a cutoff point needs to be established to determine whether the child has moved from the dysfunctional to the functional range. The second step is to calculate the RCI to determine if the child’s change from pretreatment to follow-up is not the result of measurement error. When these two criteria are combined (cutoff and RCI), the children can be classified as *Recovered* (i.e., passed both criteria), *Improved* (i.e., passed RCI criterion but not the cutoff), *Unchanged* (i.e., passed neither criterion), or *Deteriorated* [i.e., passed RCI criterion but worsened; 52].

## Results

### Baseline Comparisons

Families in the PMTO and CAU condition did not differ significantly on any of the demographic variables. It should be noted, however, that there was a trend towards significance for ADHD, with fewer children having this diagnosis in the PMTO condition [*χ* ^*2*^(3) = 7.33, *p* = .06]. Then, we checked whether there were differences in outcome measures between the PMTO and CAU group at the baseline assessment. Only one significant difference was found: children from the CAU group displayed higher levels of behavioral problems on the PDR than children in the PMTO group [*t*(119) = 2.28, *p* = .05].

### Treatment Attendance

The number of sessions families received was better documented for PMTO than for CAU. Reports on treatment attendance were available for 61 PMTO families and 18 CAU families, and these showed that PMTO families received more treatment sessions than CAU families. Families in the PMTO condition received on average 23.85 (*SD* = 9.86) treatment sessions, while families in the CAU condition received a mean of 20.50 (*SD* = 10.67) sessions. This difference was not significant [*t*(77) = 1.24, *p* = .22], and therefore we did not have to control for treatment attendance in our effect analyses.

### Effects of PMTO Versus CAU

#### Child Behavior Problems

Mean scores (and standard deviations) of children in the two treatment conditions on various CBCL and TRF scales and the PDR are shown in Table 3. First, we compared the effectiveness of PMTO and CAU on externalizing behavior problems because this was the main outcome variable. For CBCL externalizing and the PDR, a significant main effect of time was found [*F*(3, 68.87) = 14.75, *p* < .001 and *F*(1.99, 315.02) = 13.17, *p* < .001, respectively]: post hoc comparisons with Bonferroni correction showed that in both conditions, symptom levels significantly decreased from T0 to T1 after which they remained fairly stable at T2 and T3. No interaction effects of treatment condition and time were found, indicating that there were no significant differences in effectiveness between PMTO and CAU on these outcome measures [*F*(3, 112.49) < 1 and *F*(1.99, 897.57) = 2.37, *p* = .09, respectively]. For TRF externalizing, neither a main effect of time [*F*(3, 33.87) = 1.79, *p* = .17] nor an interaction effect of treatment conditions and time could be documented [*F*(2.66, 182.89) < 1].

**Table 3:** Means, standard deviations, and treatment effects for child behavior problems

	PMTO				CAU				Main effect of time	Time × treatment
	T0	T1	T2	T3	T0	T1	T2	T3		
	*M* (*SD*)	*M* (*SD*)	*M* (*SD*)	*M* (*SD*)	*M* (*SD*)	*M* (*SD*)	*M* (*SD*)	*M* (*SD*)	*F*	*F*
CBCL int	62.66 (9.38)_a_	58.39 (11.93)_b_	58.71 (17.19)_b_	57.86 (13.05)_b_	63.62 (9.01)_a_	57.62 (11.69)_b_	59.81 (14.22)_b_	57.91 (13.21)_b_	14.14*	.48
CBCL ext	70.90 (6.32)_a_	65.32 (10.70)_b_	62.99 (13.60)_b_	63.90 (12.93)_b_	71.23 (6.53)_a_	65.80 (10.90)_b_	65.35 (11.18)_b_	66.57 (10.46)_b_	14.75*	.61
CBCL total	68.60 (6.71)_a_	63.07 (9.59)_b_	61.06 (13.10)_b_	60.97 (13.77)_b_	69.32 (6.18)_a_	65.80 (11.61)_b_	64.42 (11.83)_b_	64.32 (11.00)_b_	15.10*	1.33
TRF int	56.95 (10.74)	56.56 (12.83)	53.75 (16.54)	55.31 (20.06)	58.01 (10.51)	56.76 (12.21)	53.19 (18.41)	56.58 (18.21)	1.76	.49
TRF ext	62.92 (10.27)	60.38 (12.66)	58.81 (19.08)	58.88 (14.54)	62.32 (11.59)	60.01 (12.80)	58.52 (19.83)	60.23 (15.93)	1.79	.53
TRF total	62.13 (10.00)	60.43 (9.40)	58.61 (17.42)	62.05 (15.48)	63.32 (10.79)	61.75 (10.35)	59.76 (16.01)	62.87 (15.31)	2.12	.32
PDR	17.64 (14.57)_a_	14.92 (15.87)_b_	14.14 (11.79)_b_	12.77 (14.51)_b_	21.92 (12.13)	16.00 (16.77)	15.53 (13.52)	15.52 (19.21)	13.17*	2.37

*T*-scores are presented for the internalizing, externalizing and total scales of the Child Behavior Checklist (CBCL) and the Teacher Report Form (TRF). Means with different subscripts indicate significant difference at *p* < .05 (Bonferroni corrected). PDR = Parent Daily Report

* *p* < .05

Second, treatment effects on parent and teacher rated internalizing and total problems were analyzed. The pattern of results resembled that found for the externalizing behavior problems. That is, for CBCL internalizing as well as total behavior problems, a significant main effect of time was found [*F*(2.62, 223) = 14.14, *p* < .001 and *F*(2.82, 74.87) = 15.10, *p* < .001, respectively]. Pairwise comparisons using Bonferroni correction again only revealed a significant decrease in behavior problems between T0 and T1. No interaction effects of treatment condition and time were found [*F*(2.62, 348.94) < 1 and *F*(2.82, 126.29) = 1.33, *p* = .27, respectively]. For TRF internalizing and total behavior problems, neither significant main effects nor interaction effects were found.

Cohen’s effect size *d* was calculated for the main outcome variable of the present study, i.e., parent-rated child externalizing behavior problems, in order to compare our findings to those of previous European studies.^1^ As can be seen in Table 4, the effect size of PMTO in the present study was comparable to the effect sizes for PMTO in the Norwegian studies and even somewhat higher than the effect size for PMTO as found in the investigation conducted in Iceland. With the exception of the effect size found for aggressive behavior for the control condition in the Norwegian follow-up study (Cohen’s *d* = .63), the effect size for CAU in the present study (Cohen’s *d* = .55) was generally higher than that obtained for the treatment control conditions in the Norwegian and Icelandic studies (Cohen’s *d*’s between .22 and .43).

**Table 4:** Cohen’s d in four European PMTO effectiveness studies for parent reported externalizing behavior problems

Study	Country	Follow-up assessment	Outcome measure	Cohen’s *d* PMTO	Cohen’s *d* control group
Present study	The Netherlands	18 months after baseline	CBCL ext	.73	.55
Ogden and Amlund-Hagen [20]	Norway	Post treatment	CBCL ext	.73	.43
Amlund-Hagen et al. [26]^a^	Norway	1 year after post treatment	CBCL agg	.85	.63
			CBCL del	.70	.22
Sigmarsdóttir et al. [27]^b^	Iceland	Post treatment	CBCL ext	.47	.39

*CBCL* Child Behavior Checklist, *ext* externalizing, *agg* aggressive, *del* delinquent

^a^Based on intention-to-treat (ITT) analyses; ^b ^Based on raw scores not presented in the original paper, but requested from the authors

#### Parenting Practices

Parenting practices were assessed using self-report (CWL) and structured observations (SIT). Since only the CWL subscale ‘discouraging undesirable behavior’ and the total score on this self-report measure displayed acceptable internal consistencies, only these scores were used in subsequent analyses. For discouraging undesirable behavior, a significant main effect of time was found. Parents in both conditions reported a significant increase in their employment of discouragement of undesirable behavior during the first 6 months [*F*(2.79, 53.21) = 7.21, *p* < .001]. For the CWL total score, also a significant main effect of time emerged [*F*(2.77, 79.07) = 11.05, *p* < .001] whereas the interaction of treatment condition and time did not attain significance [*F*(2.77, 978.85) = 2.15, *p* = .10]. Irrespective of treatment condition, parents reported using significantly more effective parenting skills over time during the first 12 months.

Using the SIT data in relation to the main caregiver, no significant main effects of time or interaction effects of treatment condition and time were found. For the second parent, a significant Time × Condition interaction was found for interpersonal atmosphere [*F*(3, 6942.45) = 2.74, *p* = .04]. Pairwise comparisons using Bonferroni correction showed that this interaction was apparent between T2 and T3: The second parent in the PMTO condition showed an increase in positive interpersonal atmosphere between T2 and T3, while the second parent in the CAU condition showed a decrease. No significant effect was found for child compliance [*F*(3, 18) = 1.47, *p* = .26], which was probably due to the low base rate of child problem behavior during the SITs. The mean number of coded problem behaviors during the seven tasks at baseline (T0) was .90 (*SD* = 1.54), indicating that on average children showed problem behavior in fewer than one of the seven SITs.

#### Parental Stress and Psychopathology

The mean scores (and standard deviations) of the NOSI and SCL-90-R are presented in Table 5. To assess treatment effects on parenting stress, the NOSI was used. On the parent domain, child domain, and the total score of the NOSI, a significant main effect of time was found [*F*(2.42, 45.73) = 9.60, *p* < .001, *F*(2.81, 64.14) = 12.82, *p* < .001, and *F*(2.55, 79.5) = 21.37, *p* < .001, respectively]. Post-hoc comparisons with Bonferroni correction showed that in both treatment conditions, parenting stress significantly decreased from T0 to T1 and then remained stable at T2 and T3. No interaction effects of treatment condition and time were found, which indicates there were no significant differences in effectiveness between PMTO and CAU on parenting stress.

**Table 5:** Means, standard deviations, and treatment effects for parental stress and psychopathology

	PMTO				CAU				Main effect of time	Time × treatment
	T0	T1	T2	T3	T0	T1	T2	T3		
	*M* (SD)	*M* (SD)	*M* (SD)	*M* (SD)	*M* (SD)	*M* (SD)	*M* (SD)	*M* (SD)	*F*	*F*
NOSI parent domain	148.93 (45.42)_a_	123.12 (47.43)_b_	125.74 (54.22)_b_	115.97 (51.19)_b_	148.21 (49.84)_a_	128.56 (46.95)_b_	135.35 (60.44)_b_	127.24 (53.43)_b_	9.60*	1.03
NOSI child domain	207.94 (45.69)_a_	176.85 (64.05)_b_	173.67 (91.32)_b_	168.65 (66.32)_b_	207.41 (50.92)_a_	183.19 (65.87)_b_	184.33 (75.17)_b_	185.75 (70.20)_b_	12.82*	.83
NOSI total stress	358.46 (82.68)_a_	300.50 (104.72)_b_	287.22 (107.35)_b_	283.89 (132.06)_b_	353.32 (95.63)_a_	310.31 (112.58)_b_	310.08 (113.89)_b_	313.08 (118.80)_b_	21.37*	1.72
SCL-90-R	129.81 (46.29)_a_	115.73 (34.89)_b_	118.82 (64.35)_b_	111.42 (35.22)_b_	125.86 (41.96)_a_	109.66 (31.85)_b_	117.87 (56.27)_b_	113.04 (36.25)_b_	9.71*	.63

Means with different subscripts indicate significant difference at *p* < .05 (Bonferroni corrected). *NOSI* Nijmeegse Ouderlijke Stress Index (Parenting Stress Index), *SCL-90-R* = Symptom Checklist-90 Revised

* *p* < .05

For psychopathological complaints, as measured by the SCL-90-R, there was a significant main effect of time [*F*(2.26, 145.71) = 9.71, *p* < .001]. Pairwise comparisons, using Bonferroni correction, showed that for both treatment conditions the level of psychopathology significantly decreased from T0 to T1 after which no further change was observed. Again, no significant interaction between treatment condition and time was found [*F*(2.26, 259.43) < 1].

#### Parent Satisfaction

To examine whether there was a difference in treatment satisfaction between PMTO and CAU, the scores of the WAI-S at T1 were analyzed using an independent samples *t* test. Results showed that the difference between PMTO (*M* = 43.95, *SD* = 4.67) and CAU (*M* = 42.94, *SD* = 4.75) was not significant [*t*(142) = 1.05, *p* = .16].

### Clinical Significance

Our analyses indicate that both PMTO and CAU produced statistically significant decreases of externalizing problem behavior. However, to examine whether the improvement in child behavior was also clinically significant at the individual level, the RCI was calculated for both the PMTO and CAU condition using the CBCL externalizing behavior problems scores at T0 and T3. Based on the Jacobson-Truax method [52], children could be classified as recovered, improved, unchanged, or deteriorated. The percentages of children in each category for PMTO and CAU are presented in Table 6. In total, 45.8 % of children in the PMTO condition improved against 42.8 % of the children in the CAU condition. In the PMTO group, 16.9 % of the children recovered compared with 9.7 % in the CAU group. The distribution over the four categories did not differ significantly between PMTO and CAU [*χ* ^*2*^(3) = 1.60, *p* = .66].

**Table 6:** Percentages of reliable change based on parent rated externalizing behavior problems

	PMTO	CAU
Recovered	16.9	9.7
Improved	45.8	42.8
Unchanged	25.5	33.6
Deteriorated	11.4	14.1

### Moderators

To examine whether PMTO works better for certain families than for others, we tested whether factors could be identified that moderate the effect of PMTO on the main outcome variable (i.e., CBCL externalizing). Children who were classified as recovered and improved based on the RCI (*n* = 58) were compared with children who were classified as unchanged or deteriorated (*n* = 34). We examined child variables (i.e., age, gender, severity of problem behavior at baseline, IQ), parent variables (i.e., gender main caregiver, age, ethnicity, educational level, level of parenting skills and parenting stress at baseline, job status), and family variables (i.e., single parent household, number of siblings). Only one significant result was found for the CBCL externalizing subscale at T0 [*t*(333) = 2.41, *p* < .001]. Children who showed reliable improvement in parent rated child externalizing behavior problems showed significantly more severe externalizing behavior problems at baseline (*M* = 72.19) as compared to children who did not show reliable improvement (*M* = 68.69).

## Discussion

The present study compared the effectiveness of PMTO and CAU in Dutch children who had been referred to child care organizations because of externalizing behavior problems. It was hypothesized that the PMTO treatment would result in a greater reduction of externalizing behavior problems in children, greater improvements in effective parenting skills, and less parenting stress and parental psychological complaints as compared to CAU. Furthermore, it was expected that parents in the PMTO condition would be more satisfied with the treatment than parents in the CAU condition. Finally, it was hypothesized that children who had received PMTO would more often show clinically significant change than children treated with CAU.

In contrast with our expectations, the results revealed no statistically significant differences in effectiveness between PMTO and CAU on the primary treatment outcome measures of parent-reported externalizing behaviors. That is, children in both conditions showed a significant decrease in CBCL externalizing and PDR scores within the first 6 months of treatment, after which symptom levels remained fairly stable. For parent-rated internalizing and total behavior problems, a similar pattern was found: in both treatment conditions significant decreases were found during the first 6 months, but no evidence was obtained that children in the PMTO condition fared better than those who received CAU. The fact that internalizing symptoms were also reduced following interventions which essentially target externalizing problems, suggests that either non-specific treatment factors were at work or that both interventions were capable of tackling a process underlying both types of problems. However, a reduction of internalizing behavior problems is a common finding in studies evaluating parent training programs for externalizing behavior problems [e.g., 54, 55]. No effects were found for teacher-reported behavior problems. One explanation for this unexpected result might be that children’s behavior problems are less apparent at school and that, therefore, change was less noticeable. Indeed, the data indicated that teachers in general reported less problem behavior as compared to parents. Alternatively, it is also possible that the positive treatment effects did not generalize to the school setting and that the interventions are only effective in the context where they have been implemented (i.e., at home).

The finding that PMTO did not result in a greater decrease of externalizing behavior problems than CAU, is in contrast with the results of previous studies showing a superiority of PMTO over control interventions [e.g., 11, 18, 24]. However, it is important to note that most of the earlier studies that have been conducted in the United States compared PMTO to a waiting list control condition. The families included in the control condition of our study also received a proper treatment, which turned out to be rather effective in reducing children’s externalizing problems. Our findings seem to be more in line with the results of two PMTO effectiveness studies conducted in Norway and Iceland, which also included a control group that received an alternative treatment [20, 27]. The Norwegian study demonstrated that PMTO initially resulted in a larger decrease in problem behaviors than CAU, but also found that this difference was no longer significant at one-year follow-up [26]. In the Icelandic study, PMTO produced a better treatment effect than CAU on children’s social skills, but not on behavior problems [27]. It is noteworthy that the effect size of CAU in our study was generally larger than that obtained in the other studies, which indicates that the general treatment offerings for children with externalizing problems in The Netherlands appears to be of good quality. This probably is a result of the fact that many psychologists in this country are trained to apply cognitive-behavioral techniques, which seem to be an important ingredient of effective interventions for children with externalizing problems [56]. In addition, PMTO is not the only treatment for externalizing behavior problems in The Netherlands that was not more effective than CAU [e.g., Triple P; 57, 58].

Contrary to our expectations, no significant differences between PMTO and CAU were found with regard to the application of effective parenting skills. Only three significant findings on parenting skills emerged. The first one was that parents in both conditions reported a significant increase in self-reported discouragement of undesirable behaviors over time. This suggests that parents in general became more responsive to the misbehaviors of their child. Second, parents reported an increase in their overall use of self-reported effective parenting practices over time. Third, when analyzing the behavioral observation data on parenting behavior, neither PMTO nor CAU showed significant improvement in parenting skills over time for the main caregiver. However, a difference between PMTO and CAU was found for interpersonal atmosphere of the second caregiver. The second caregiver who had received PMTO demonstrated a more positive interpersonal atmosphere over time as compared to the second caregiver who had received CAU.

PMTO, as derived from the SIL model, assumes that the reduction of problematic child behavior is mediated by improvements in parenting skills. In particular effective discipline is thought to be an important target mechanism involved in the elimination of child externalizing problems [20, 59]. Note, however, that this could only be demonstrated with the self-report measure in our study, and this may be due to several reasons. First of all, the observational tasks we used did not elicit particularly high levels of negative behaviors in the child, so parents hardly had to discipline their child during these assessments. Even at baseline, when children were expected to show clear signs of externalizing behavior, the frequency of such problems was less than one out of the seven observation tasks. A second explanation concerns the (un)reliability of the observations. It should be noted that not all parenting scales had satisfactory inter-rater reliability (e.g., discipline). Further, one could argue that the SITs were too well-structured for the oppositional-defiant behavior of the child and the accompanying parenting responses to emerge, which of course questions the ecological validity of our observation measure. Still, it eludes us why our children ‘behaved so well’ during the tasks, because we used tasks very similar to the ones used in the original studies [e.g., 18, 24]. One difference is that our SITs were typically administered in a plain room with few distractors, while in the original studies toys and other distractors were available and present in the room. Similar points of critique can be raised regarding the self-report measure of parenting skills. The internal consistency of five out of six subscales of the CWL was unsatisfactory, and there are data that seriously question the validity of this measure [60]. Nevertheless, the two reliable scales of the CWL (discouraging undesirable behavior and CWL total score) did show a positive treatment effect.

In both conditions, significant reductions of parenting stress and parental psychopathology within the first 6 months were found, with no significant differences observed between PMTO and CAU. These results indicate that parents generally felt better as a result of both types of treatment. Apparently, the improvements in their child’s behavior make parents feel less stressed during daily interactions with their child, which may well translate into an overall improved sense of well-being, although the direction of this effect may also be reversed: receiving treatment may boost parental self-efficacy and well-being, which in turn has a positive impact on children’s behavior [e.g., 28, 61].

Although it was expected that parents in the PMTO condition would be more satisfied with the treatment compared to CAU, this was not confirmed by our results. Parents receiving PMTO were just as satisfied as parents receiving CAU. However, it may well be that treatment satisfaction is intimately related to treatment effectiveness. Since PMTO appeared to be equally effective as CAU, it was not surprising that parents were also comparably satisfied with both types of interventions.

Not all children profited equally from the PMTO and CAU interventions. A detailed analysis (combining reliable change and clinical cut-off) indicated that 17 % of the children within the PMTO group recovered and 46 % showed reliable improvement in externalizing behavior. In comparison, in the CAU condition 10 % of the children recovered and 43 % reliably improved. Yet, these differences between PMTO and CAU in reliable change were not significant, implying that PMTO did not yield more clinically significant change, which is in accordance with our results discussed above. To determine if some children benefited more from PMTO than others, several possible moderators were examined. Only one moderator effect was found: children who improved or recovered had significantly higher parent-rated externalizing behavior problems at baseline as compared to children who did not change or worsened. Thus, especially children with serious externalizing behavior problems appeared to benefit more from PMTO. This result is probably due to the fact that there was simply more room for improvement for these children. Possibly, more moderator effects would have been found when using only the recovered and deteriorated children in the comparison. However, in the present study, these subgroups were too small to conduct such analyses.

A number of limitations of the present study should be mentioned. First, although the study was originally designed as a RCT, due to practical constraints, we had to continue as a quasi-experimental investigation about halfway through the study. This also resulted in an unequal number of families in the PMTO and CAU conditions. Second, we did not have information about the actual number of treatment sessions that families in both conditions received, and therefore we were not able to control for treatment exposure. Also, information on medication use was not systematically documented. Third, as described above, the assessment of parenting practices appeared to be quite problematic, and this appeared true for both the self-report measure (CWL) and the observations (SITs). With regard to the observational index, an additional shortcoming was that coders not always remained blind to treatment condition and time-of-assessment (i.e., T0, T1, T2, T3), because of (unwanted) comments about the treatment made by parents or the assessor during the interaction tasks.

In spite of these limitations, we can conclude that a PMTO intervention produced positive effects in a clinically referred sample of children with externalizing problems in the Netherlands. More precisely, this treatment was effective in reducing children’s problem behaviors (even showing a quite large effect size), increasing the use of self-reported effective parenting practices, and reducing parenting stress and psychopathological symptoms of the parents, albeit no more effective than CAU. For both conditions, the improvements were most evident during the first 6 months of the study and remained stable until 18 months after baseline. Although many effects of the present study were in favor of PMTO and comparable to the effects of PMTO in other European countries, CAU in our study appeared to perform better than the control conditions in most other studies. It is remarkable to note that many of the CAU interventions performed within the Dutch youth care system also include the therapeutic ingredients, such as the use of ‘time out’ for disciplining and rewarding desired behaviors, that are considered important in PMTO. In a future study, the cost-effectiveness of PMTO will be compared to CAU. Annual youth service costs have been rising steadily over the past decade in The Netherlands, and a cost-benefit analysis will provide policy makers and insurance companies with quality information to guide decision-making, in the interest of young children, families and society at large.

## Summary

The present study examined whether parent management training—Oregon model (PMTO) is more effective as a treatment for children with externalizing behavior problems in The Netherlands than Care As Usual (CAU). Clinically referred children (N = 146) aged 4–11 years and their parents were included in this research of which 91 received PMTO and 55 CAU. Families were assessed at four time points: at pretreatment, and after 6, 12, and 18 months. Results showed no statistically significant differences in the effectiveness of the two interventions. Both treatment conditions were effective in reducing children’s problem behaviors, increasing the use of (self-reported) effective parenting practices, and alleviating parenting stress and psychopathological symptoms of the parents. The improvements were most evident during the first 6 months of the study and remained stable until the 18-months follow-up assessment. Additionally, we found that especially children with serious externalizing behavior problems at baseline benefited from PMTO. Comparing the effect size of PMTO delivered in The Netherlands with previous PMTO effectiveness studies in Norway and Iceland, we demonstrated that PMTO had a similar, large effect size as shown in previous studies. From these findings it can be concluded that PMTO is effective in a clinically referred sample of children with externalizing problems in the Netherlands, although it seems to be no more effective than CAU.

## Footnotes

1 We limited our comparison to European studies because these studies were similar to our study in terms of design, sample, and culture.
